# Editorial: Congruities between cancer and infectious diseases: Lessons to be learned from these distinct yet analogous fields

**DOI:** 10.3389/fcimb.2022.1072022

**Published:** 2022-12-07

**Authors:** Shyamala Thirunavukkarasu, Prasanna K. Santhekadur

**Affiliations:** ^1^ Department of Molecular Microbiology, Washington University in St. Louis School of Medicine, St. Louis, MO, United States; ^2^ Department of Biochemistry, Center of Excellence in Molecular Biology & Regenerative Medicine, Jagadguru Sri Shivarathreeshwara (JSS) Medical College, JSS Academy of Higher Education and Research, Mysore, Karnataka, India

**Keywords:** cancer, infectious diseases, bacteria, viruses, inflammation

It is a very well-established fact that cancer is a multifactorial disease involving innumerable complex cellular signaling pathways (Wu et al., 2018). These pathways are mainly regulated by oncogenes and tumor suppressor genes (Lee and Muller, 2010). Recent studies show that microRNAs (miRNAs) and associated RNA-induced silencing complex (RISC) also play a major role in oncogenesis (Yoo et al., 2011; Santhekadur and Kumar, 2019; Santhekadur et al., 2012). Infectious diseases are serious health disorders, and they are mainly caused by various unicellular or multicellular organisms such as bacteria, viruses, and fungi (Prasad et al., 2022; José et al., 2020). Infectious human viruses like human papillomavirus (HPV) cause cervical cancer; Epstein–Barr virus (EBV) is known to cause nasopharyngeal cancer and Burkitt lymphoma. Liver-associated human viruses like hepatitis B virus (HBV) and hepatitis C virus (HCV) cause hepatitis and induce hepatocellular carcinoma (Liao, 2006). Bacteria, namely, *Helicobacter pylori*, are also known to cause gastric cancer (Prasad et al., 2022). Some of the fungal secondary metabolites and toxic chemicals are known to trigger mutations and initiate cancer growth and development (Ekwomadu et al., 2022). Fungal origin environmental toxins like aflatoxin and ochratoxin are known carcinogens and activate inflammation and carcinogenesis (Shiragannavar et al., 2021; Vamadevaiah and Santhekadur, 2022) (Felizardo and Câmara, 2013). Inflammation is a major contributor and a well-known hallmark of almost all of the infectious diseases and carcinogenesis. Inflammatory cytokines like tumor necrosis factor alpha (TNF-α) and transcription factor nuclear factor kappa B (NF-κB) act as master regulators of inflammation-associated infectious diseases and cancer (Santhekadur et al., 2012; Prasad et al., 2022). Therefore, although distinct, there exists a strong congruity between cancer and infectious diseases. This special collection of original research and review articles gave us new insights on the microorganism-associated cancer development and progression and also beneficial effects of fungal secondary metabolites in cancer therapeutics.

This Research Topic contains a study by Li et al. which showed that the reactivation of cytomegalovirus (CMV) or EBV is closely related to poor hematopoietic stem cell transplantation (HSCT) outcomes. CMV is strongly correlated with different types of cancer initiation, including breast, colon, prostate, and ovarian cancer. EBV infection also increases a person’s susceptibility to certain types of cancer such as nasopharyngeal cancer, Burkitt lymphoma, and Hodgkin lymphoma. This retrospective study strongly interprets the dangerous effects of these two viruses even in patients who have undergone HSCT.

The ubiquitin proteasome pathways are involved in protein degradation and recycling; they also aid in amino acid homeostasis (Uriarte et al., 2021). The review article by Ignatz-Hoover et al. revealed that the proteasome acts as a highly promising appropriate therapeutic target to combat infectious diseases and different types of cancers. Dolatabadi et al. showed the presence of intracellular highly adherent-invasive *Escherichia coli* among different stages of the disease, family background, and history in treated colorectal cancer patients in Iran. Although *E. coli* is a harmless bacterium and is an important part of a healthy human gut microbiota, this study clearly shows the pathogenic nature of a few strains of *E. coli* in infectious diseases and cancers. A study by Varli et al. showed that acetonic extract and secondary metabolites from endolichenic fungus (*Nemania* sp. EL006872) exhibited immune checkpoint inhibitory activity especially in lung cancer cells. This study also shows the potential importance of fungal secondary metabolites, and they can be used as promising therapeutic molecules in cancer therapy. This article indirectly shows that these microbial metabolites not only cause inflammation and cancer but also carry importance in cancer immunotherapy. Another study by Wang et al. also showed how fucoxanthin prevents breast cancer growth and metastasis by stopping circulating tumor cell adhesion and transendothelial migration. This study clearly shows the anticancer potential of fucoxanthin in which it inhibited the most important hallmarks of breast cancer by suppressing circulating tumor cell adhesion and transendothelial migration. Interestingly, this fucoxanthin is a xanthophyll carotenoid most abundantly present in microorganisms like macroalgae and multicellular organisms like brown seaweeds.

All of these elegant studies clearly show the existence of congruities between cancer and infectious diseases. Furthermore, additional experimental evidence and studies give more knowledge to understand these distinct yet analogous fields.

## Author contributions

All authors listed have made a substantial, direct, and intellectual contribution to the work and approved it for publication.
